# Variable expression and silencing of CRISPR-Cas9 targeted transgenes identifies the *AAVS1* locus as not an entirely safe harbour

**DOI:** 10.12688/f1000research.19894.2

**Published:** 2020-07-10

**Authors:** Jamie R. Bhagwan, Emma Collins, Diogo Mosqueira, Mine Bakar, Benjamin B. Johnson, Alexander Thompson, James G.W. Smith, Chris Denning

**Affiliations:** 1 Department of Stem Cells, Tissue Engineering and Modelling, Centre for Biomolecular Sciences, University of Nottingham, Nottingham, NG7 2RD, UK; 2 Faculty of Medicine and Health Sciences, Norwich Medical School, University of East Anglia, Norwich Research Park, Norwich, NR4 7UQ, UK

**Keywords:** Human induced pluripotent stem cells; CRISPR/Cas9, stem-cell derived cardiomyocytes, stem-cell derived haematopoietic cells, AAVS1 safe harbour, gene targeting, silencing

## Abstract

**Background:** Diseases such as hypertrophic cardiomyopathy (HCM) can lead to severe outcomes including sudden death. The generation of human induced pluripotent stem cell (hiPSC) reporter lines can be useful for disease modelling and drug screening by providing physiologically relevant *in vitro* models of disease. The *AAVS1* locus is cited as a safe harbour that is permissive for stable transgene expression, and hence is favoured for creating gene targeted reporter lines.

**Methods**: We generated hiPSC reporters using a plasmid-based CRISPR/Cas9 nickase strategy. The first intron of *PPP1R12C*, the *AAVS1* locus, was targeted with constructs expressing a genetically encoded calcium indicator (R-GECO1.0) or HOXA9-T2A-mScarlet reporter under the control of a pCAG or inducible pTRE promoter, respectively. Transgene expression was compared between clones before, during and/or after directed differentiation to mesodermal lineages.

**Results**: Successful targeting to *AAVS1* was confirmed by PCR and sequencing. Of 24 hiPSC clones targeted with pCAG-R-GECO1.0, only 20 expressed the transgene and in these, the percentage of positive cells ranged from 0% to 99.5%. Differentiation of a subset of clones produced cardiomyocytes, wherein the percentage of cells positive for R-GECO1.0 ranged from 2.1% to 93.1%. In the highest expressing R-GECO1.0 clones, transgene silencing occurred during cardiomyocyte differentiation causing a decrease in expression from 98.93% to 1.3%. In HOXA9-T2A-mScarlet hiPSC reporter lines directed towards mesoderm lineages, doxycycline induced a peak in transgene expression after two days but this reduced by up to ten-thousand-fold over the next 8-10 days. Nevertheless, for R-GECO1.0 lines differentiated into cardiomyocytes, transgene expression was rescued by continuous puromycin drug selection, which allowed the Ca ^2+^ responses associated with HCM to be investigated *in vitro* using single cell analysis.

**Conclusions:** Targeted knock-ins to *AAVS1* can be used to create reporter lines but variability between clones and transgene silencing requires careful attention by researchers seeking robust reporter gene expression.

## Introduction

A key consideration for targeted gene delivery in human induced pluripotent stem cells (hiPSCs) is the genomic location at which to insert the exogenous DNA sequence to maximise transgene expression and limit disruption of critical endogenous genes and their function. To this end, a number of chromosomal locations that are amenable to integration have been exploited. These regions of the genome are commonly referred to as safe harbour loci, and often share some common properties such as limited disruption to endogenous genes, low proximity to oncogenes and a chromatin structure that is not prone to epigenetic silencing ^1, 2^.

Examples of previously utilised genomic safe harbour loci include the chemokine (C-C motif) receptor 5 ( *CCR5*) gene ^3, 4^, the human orthologue of the mouse Rosa26 locus ( *hROSA26*) ^5^, and a region within intron 2 of the Citrate Lyase Beta-Like ( *CLYBL*) gene ^6^.

The *AAVS1* locus is an area of chromosome 19 (position 19q13.42) that has been found to be a common integration site for exogenous DNA delivered to cultured cells with adeno-associated virus (AAV) ^7, 8^. Integration into this site is associated with only limited disruption of endogenous genes. The phosphatase 1 regulatory subunit 12C ( *PPP1R12C*) gene codes for a protein with a poorly defined function, and its first intron is disrupted by integration into the *AAVS1* site, with no observed deleterious consequences in targeted human pluripotent stem cells (hPSCs) ^2, 9^. DNA sequences inserted at this location are supposedly protected by endogenous insulator regions ^10^. These insulators are considered to contribute to maintaining an open chromatin conformation at the *AAVS1* locus, reducing the likelihood of transgene silencing compared to other safe harbour loci such as *CCR5* ^4, 11^. However, some reports of DNA methylation dampening transgene expression in both hPSC-derived hepatocytes ^12^ and iPSC-derived myeloid progenitors ^32^ raise questions on whether a ‘perfect’ safe harbour locus exists. Despite this, *AAVS1* has remained popular for gene targeting ^13– 16^.

We sought to utilise CRISPR/Cas9 nickase to target the *AAVS1* locus in hiPSCs and introduce a genetically encoded calcium indicator, R-GECO1.0, to enable live Ca ^2+^ imaging in hiPSC-derived cardiomyocytes (hiPSC-CMs) ^18, 19^. This was performed in genome engineered isogenic hiPSC lines we previously described to model the condition, hypertrophic cardiomyopathy (HCM). These included a trio of lines harbouring a c. *MYH7* ^C9123T^ mutation ^20^ and a duo harbouring a c. *ACTC1* ^G301A^ mutation ^21^.

In addition, CRISPR Cas9 targeting of the *AAVS1* locus was used to target a doxycycline-inducible HOXA9-T2A-mScarlet cassette into hiPSCs with the aim of modulating HOXA9 during haematopoietic differentiation. HOXA9 is a transcription factor regulated spatio-temporally during haematopoietic or cardiac development ^22^ and the aim was to examine if controlled supplemental expression of HOXA9 resulted in more efficient production of mature cells.

We found that, far from being a safe harbour locus, *AAVS1* integration associated with transgene expression that varied between clones and/or was silenced during directed differentiation towards both haematopoietic cells and cardiomyocytes. This suggests that silencing at the *AAVS1* locus is not limited to the endoderm lineage as previously described ^12^. Nevertheless, by altering our methods from bulk population analysis to single cell confocal laser line scan microscopy, we used the hiPSC-CMs expressing R-GECO1.0 to investigate the impact of HCM mutations on Ca ^2+^ transients. Abnormalities were found in both HCM-associated mutations c. *MYH7* ^C9123T^ and c. *ACTC1* ^G301A^, and this phenotype was successfully rescued with drug treatment. This demonstrates an *in vitro* alternative to some aspects of drug testing on animal models of HCM. Finally, we conclude that the *AAVS1* locus cannot be considered a true safe harbour. Researchers seeking to target this locus should check clones for transgene expression status both in hiPSCs and in differentiated progeny.

## Methods

### Ethical statement

Informed patient consent was obtained for all patient-derived hiPSC samples to be used for research purposes. Isolation and use of patient fibroblasts was approved by the Nottingham Research Ethics Committee (License 09/H0408/74), and sample collections are registered with the UK Clinical Research Network under project 8164.

### hiPSC culture and differentiation

All cell culture experiments were performed in a type II Biological Safety Cabinet, and cells were incubated in a humidified incubator at 37°C and 5% CO _2_. hiPSCs were routinely maintained in E8 medium on 1:100 Matrigel (Corning #356235) coated plastic ware (Nunc). Cells were passaged every three days by washing once with Ca ^2+^/Mg ^2+^-free Phosphate Buffer Saline (PBS, Gibco #14190-094), followed by incubation with TrypLE for four minutes. Subsequently, hiPSC were resuspended in E8 supplemented with 10 μM Y-27632 (ROCKi, Tocris Bioscience #1254/10) and seeded into new Matrigel-coated flasks at approximately 20000 cells/cm ^2^. Medium was changed every day.

hiPSC differentiation to cardiomyocytes was performed as previously described ^20, 21^. Briefly, culture vessels were seeded at approximately 20–40 thousand cells / cm ^2^, followed by a Matrigel™ overlay step two days later, supplemented with 1 ng/ml BMP4 [R&D #314-BP-050]. 16 hours later, medium was changed to StemPro™34- Serum Free Medium [SP34, Gibco #10639011], supplemented with 8 ng/ml Activin A (ActA, LifeTechnologies #PHC9564) and 10 ng/ml BMP4. After 48 hours medium was changed to RPMI B27 without insulin (LifeTechnologies #A1895601), with 10 μM KY0211 (R&D #4731) and 10 μM XAV939 (R&D #3748). 48 hours later, medium was changed to RPMI B27 (LifeTechnologies #0080085-SA) with 10 μM KY0211 and 10 μM XAV939. Thereafter, medium was changed every 2–3 days with fresh RPMI B27 until day 15 of differentiation, when hiPSC-CMs were dissociated using collagenase ^23^, re-plated, and kept in RPMI B27 for another week until phenotypic assays were performed.

hiPSC differentiation to haematopoietic cells was performed by passaging hiPSCs using Gentle Cell Dissociation Reagent (GCDR; Stem Cell Technologies #07174) and Corning cell scrapers (Sigma #CLS3008) when colonies had compacted and wells were 70–80% confluent. Differentiation was performed using STEMdiff™ Haematopoietic Kit according to the supplied protocol (Stem Cell Technologies #05310). Briefly, hiPSCs were dissociated as cell aggregates with GCDR for 7–10 minutes at room temperature, followed by scraping. Cell aggregates (50–200 µm in diameter) were seeded at different ratio densities in E8 media. The following day, only wells that contained 16–40 colonies >50 µm in diameter were selected to continue with differentiation and media was changed to Media A (day 0 of differentiation). On day two, a half Media A change was performed. On day three, media was changed to Media B and half Media B changes were performed on days five, seven and 10. On day 12, suspension cells were harvested for downstream analysis.

### CRISPR-Cas9 gene targeting of the *AAVS1* locus

In order to target the *AAVS1* locus in hiPSCs, a targeting vector was constructed containing either the CAG-R-GECO1.0-IRES-Puro cassette ^18^ or the doxycycline-inducible HOXA9-T2A-mScarlet-CAG-G418 cassette flanked on each side with 1 kb of homology to the *AAVS1* locus ^24^. 1 µg of *AAVS1* targeting vector was transfected into 1 × 10 ^6^ hiPSCs, with 500 ng of each *AAVS1* guide RNA pU6 vector and 1 µg of hCas9 D10A nickase plasmid using an Amaxa 4D system (Lonza) according to the manufacturer’s instruction. 24 hours after transfection, the medium was supplemented with 0.3 µg/ml puromycin (Life Technologies #A1113802) or 50 µg/ml Geneticin™ (Life Technologies #10131027) depending on the drug selection cassette for positive selection of clones up to 10 days post-transfection. Drug-resistant clones were then isolated using 0.5 mM EDTA and expanded. Clones were then genotyped using polymerase chain reaction (PCR) on genomic DNA using Phusion® polymerase (NEB Cat# M0530S) and the primers given in Table 1. PCR cycle parameters were 95°C for 2 minutes, 60–64°C for 30 seconds and 72°C for 60 seconds, with a final elongation step of 72°C for 10 minutes.

**Table 1.** Primers used for PCR screening of *AAVS1* integration.

PCR screen	Forward primer sequence (5’ → 3’)	Reverse primer sequence (5’ → 3’)	Annealing temperature
5’ integration screen (Outside *AAVS1* Left Arm Homology – CAG promoter)	TCCCCTCTTCCGATGTTGAG	TGGGCTATGAACTAATGACCCCG	64°C
3’ integration screen (IRES/Puromycin – Outside *AAVS1* Right Arm Homology)	AGCGTATTCAACAAGGGGCT	ACCCCGAAGAGTGAGTTTGCC	62°C
Biallelic targeting screen (Inside *AAVS1* Left Arm Homology – Inside *AAVS1* Right Arm Homology)	ATGCCGTCTTCACTCGCTGG	GGGGCTTTTCTGTCACCAATCC	64°C

### Immunocytochemistry

Dissociated hPSC-CMs or hPSCs were cultured in vitronectin- or MT-coated 96-well plates (CellCarrier, Perkin Elmer #6005550), respectively, at approximately 50K cells/cm ^2^ as described above. Cells were washed with PBS and fixed in 4% Paraformaldehyde (PFA, Sigma) at room temperature (RT) for 15 minutes. Afterwards, cells were washed in 0.1% Tween-20 (Fisher Scientific) in PBS, permeabilized with 0.1% Triton-X (Sigma) in PBS for 15 min at RT, and incubated with 4% goat serum (Sigma) in PBS (blocking solution) for one hour at RT to prevent unspecific antibody binding. Subsequently, primary antibody incubation was performed overnight at 4°C in blocking solution at the following dilutions: mouse monoclonal anti-OCT4-1:200 (Santa Cruz Biotechnology Cat# sc-5279, RRID:AB_628051), rabbit polyclonal anti-RFP-1:1000 (Abcam Cat# ab124754, RRID:AB_10971665), mouse monoclonal anti-α-actinin-1:800 (Sigma-Aldrich Cat# A7811, RRID:AB_476766). Thereafter, samples were washed three times with 0.1% Tween-20 in PBS and incubated with Alexa Fluor secondary antibodies (Life Technologies) in blocking solution for one hour at RT. Afterwards, cells were washed with 0.1% Tween-20 in PBS for 3x five minutes, followed by nuclei and/or whole cell counterstaining with 0.5 µg/ml DAPI (Sigma #D9542) or Cell Mask (1:10000, Invitrogen #H32721) in PBS, respectively, for 30 minutes at RT. Samples were subsequently washed and stored at 4°C in PBS until automated image acquisition was performed in the Operetta™ high-content imaging system (PerkinElmer) and analysed using Harmony high-content imaging analysis software.

### Live imaging mScarlet expression

HOXA9 and mScarlet expression was induced with the addition of 1 µg/ml doxycline every 48 hours. Live imaging of mScarlet fluorescence in differentiating hiPSCs was performed using Operetta™ high-content image analysis every two days. All images were taken using a 20x objective. Brightfield images were taken using 100 ms exposure time. mScarlet imaging was performed using 400 ms exposure and an excitation wavelength of 520–550 nm and an emission wavelength of 560–630nm. Data analysis was performed using Columbus™ software (PerkinElmer) to identify and quantify cell regions expressing mScarlet fluorescence.

### Gene expression analysis by qPCR

Real-time qPCR reactions were performed using TaqMan ^®^ Gene Expression Assays (Applied Biosystems) following manufacturer’s instructions. Briefly, Taqman ^®^ mastermix (#4369016) including the *HOXA9* probe ^25^ was added to a MicroAmp Fast 96-well plate (#4346907). Subsequently, cDNA samples (from initial 500 ng of reverse-transcribed RNA) were added to the plate, which was thereafter sealed with a film (#4311971). Amplification was performed in ABI 7500 Real-Time PCR system (Applied Biosystems). Cycle conditions were 50°C for 2 minutes, 95°C for 10 minutes followed by 40 cycles of 95°C for 15 seconds and 60°C for 1 minute.). Normalisation was performed using the housekeeping gene *B2M* or *PP1A*. Relative quantification was calculated using the ΔΔCT method ^26^ in Microsoft Excel.

### Confocal analysis and ClampFit identification of abnormal Ca ^2+^ transients

hiPSCs were differentiated as previously described ^20, 21^ in RPMI B27 without phenol red and dissociated on day 15 by collagenase treatment. On day 30, hiPSC-CMs were seeded at a density of 150,000 cells per well in vitronectin-N coated MatTek dishes. Intracellular Ca ^2+^ transient measurements were made using an LSM 880C confocal microscope (Carl Zeiss) in the line-scan mode as previously described ^27^. CMs were located using a 40x oil objective and a longitudinal line was drawn across a single CM. The R-GECO fluorophore was excited with a 561 nm laser at 0.8% power, with a detection range of 579 – 639 nm. Line-scan images were taken every 75 milliseconds, with a pixel dwell time of 4.12 µsec, for a total of 4000 cycles resulting in a five minute scan. CMs were kept at 37°C and 5% CO _2_ and allowed to spontaneously beat throughout data acquisition.

Confocal line scan images were analysed in FiJi software, a version of ImageJ (National Institute of Health). The average fluorescence intensity of each line was calculated against time to give a confocal line-scan trace. Using the ‘multi kymograph’ function, a corresponding kymograph image was produced. In order to calculate beat rate and arrhythmic events, data was fed into pClamp software (Molecular Devices). Baselines were adjusted to account for photobleaching and Ca ^2+^ transients were counted and analysed using the ‘event detection’ function. Using the event viewer, any Ca ^2+^ transients that did not return to baseline and gave a ‘double peak’, or did not return to at least 75% of the previous Ca ^2+^ transient amplitude, were considered ‘abnormal’, as described in 21.

### Statistical analysis

All data presented as mean with standard deviation unless otherwise stated. Statistical analysis of multiple data sets was performed using GraphPad software (version 7.04).

For multiple comparisons between data sets a one-way ANOVA with Tukey’s multiple comparison test was chosen. For comparing multiple data sets to a single control column, one-way ANOVA with Dunnett’s multiple comparison test was chosen. Significance tests were based on p-values as follows: * p < 0.05; ** p < 0.01; *** p < 0.001; **** p < 0.0001.

## Results

### Knock-in of transgenes into the *AAVS1* locus

Our overarching goal was to create two isogenic sets of hiPSC lines in order to study Ca ^2+^ handling in the context of *in vitro* models of the disease HCM. One isogenic trio comprised lines that were originally wild-type ( *MYH7* ^WT/WT^), and then CRISPR Cas9 edited to generate heterozygous ( *MYH7* ^WT/MUT^) and homozygous ( *MYH7* ^MUT/MUT^) mutants for the c. *MYH7* ^C9123T^ mutation ^20^. The other comprised a pair that were heterozygous originally patient-derived ( *ACTC1* ^WT/MUT^) and corrected ( *ACTC1* ^WT/WT^) for the c. *ACTC1* ^G301A^ mutation ^21^. for the *c. ACTC1* ^G301A^ mutation and CRISPR Cas9 corrected ( *ACTC1* ^WT/WT^) ^21, 28^.

**Figure 1.**
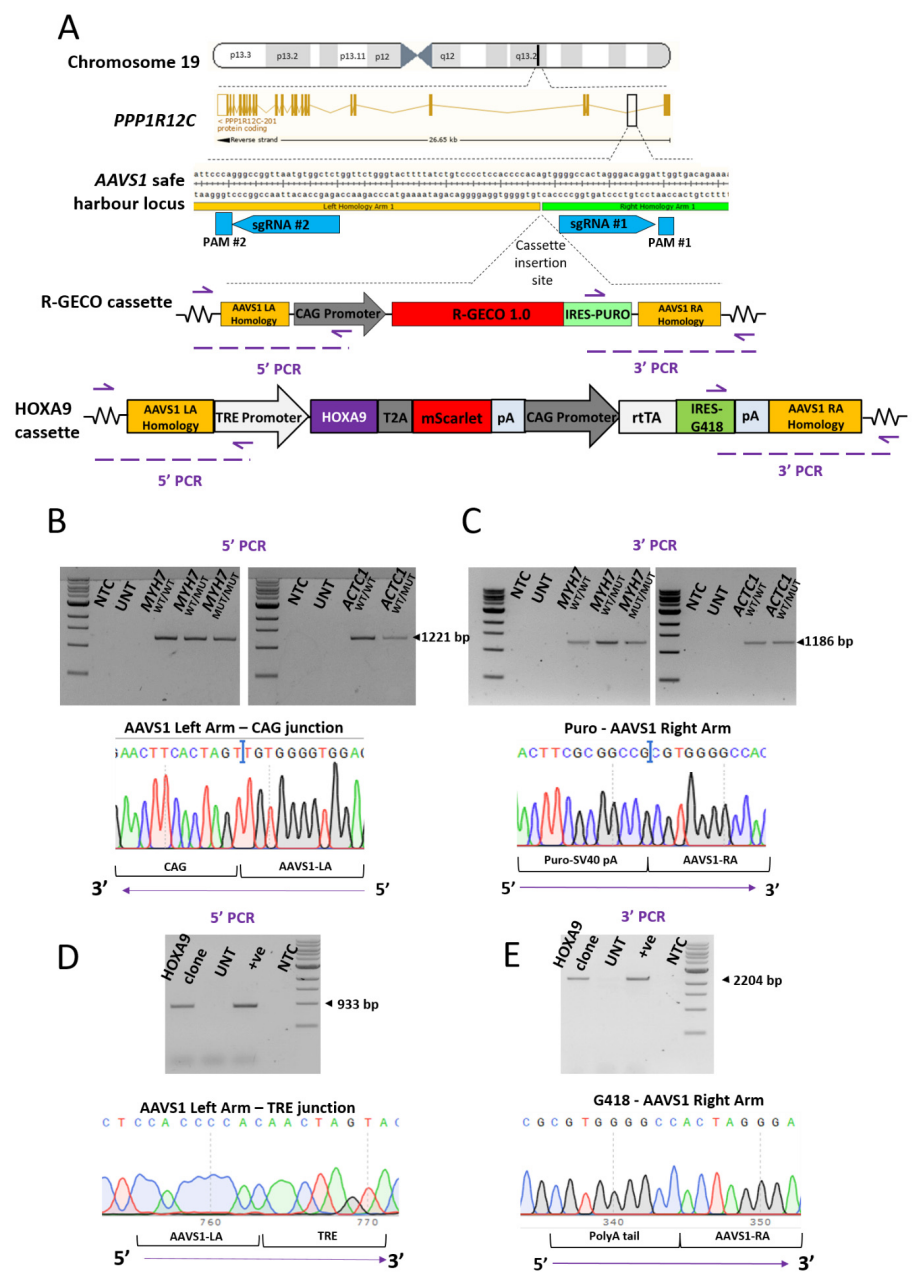
Generation of *AAVS1*-targeted hiPSC clones in an isogenic *MYH7* C9123T background and an isogenic *ACTC1* G301A background. ( **A**) Schematic illustrating the chromosomal location of the *AAVS1* safe harbour locus. This site was targeted using two sgRNAs in a CRISPR Cas9 nickase strategy. PAM site #1 was silently mutated (G→C) in the targeting construct to prevent it being cut by Cas9 nuclease during targeting. The inserted cassette consists of R-GECO1.0 IRES-Puromycin driven by the CAG promoter. This is flanked on each side by 1 kb of homology to the *AAVS1* locus. In ( **B**) and ( **C**) confirmatory 5’ and 3’ targeting PCR screen is shown using genomic DNA isolated from the *MYH7* C9123T RGECO1.0 isogenic trio (left) and the *ACTC1* G301A RGECO duo (right) hiPSCs. Correct 5’ targeting is indicated with a 1221bp product, with sequencing confirming the fidelity of the junction between the *AAVS1* left arm homology and the start of the CAG promoter. Correct 3’ targeting is indicated with an 1186bp product, with sequencing confirming the fidelity of the junction between the puromycin-SV40 pA sequence and the *AAVS1* right arm homology. ( **D**, **E**) Confirmatory PCR and sequencing of hiPSC clones to check 5’ and 3’ targeting of the *AAVS1* locus with the HOXA9-T2A-mScarlet cassette.

**Figure 2.**
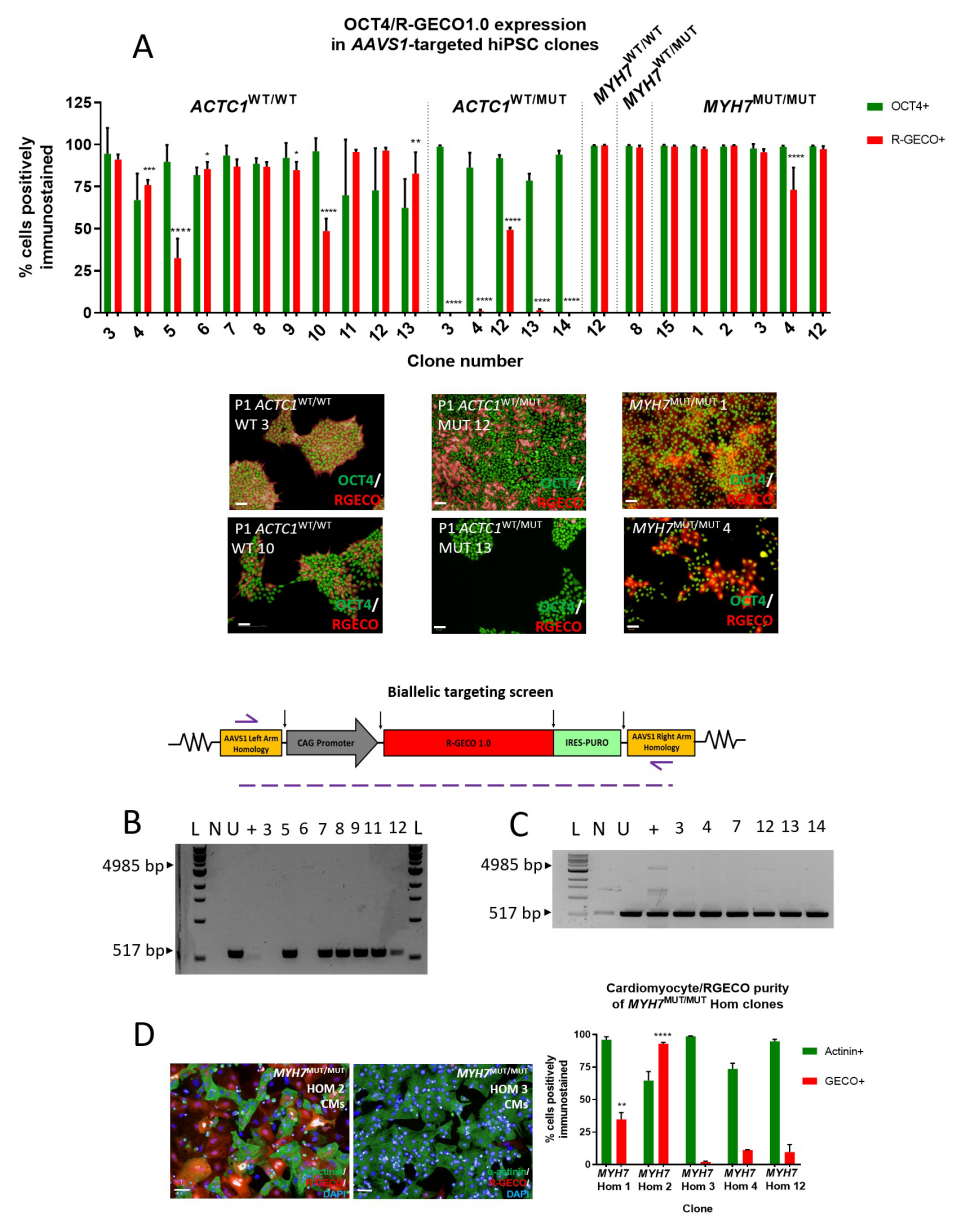
Immunocytochemistry-based screening of *AAVS1*-targeted clones showing differential expression of R-GECO1.0 between and within cell lines. ( **A**) *AAVS1* targeted hiPSC clones dual-stained for OCT4 (green) and R-GECO (red) to find the highest R-GECO-expressing clone within the five cell line genotypes. High content image analysis identified that *ACTC1* ^WT/WT^ clone 3 had the highest percentage of pluripotent (94.53% OCT4+) and R-GECO (91.06% ±1.73%) hiPSCs. *ACTC1* ^WT/MUT^ clone 12 clone had the highest expression of R-GECO (49.37% ±1.33%). Mean ±SD, n = 3 wells. One-way ANOVA with Dunnett’s multiple comparison test, * p ≤ 0.0259; ** p < 0.0045; **** p < 0.0001. Scale bars = 50 µm. ( **B**) Biallelic targeting PCR screen on isolated gDNA from targeted *ACTC1* ^WT/WT^ hiPSC clones showing homozygous clones failure to generate the 517bp PCR product. ( **C**) Biallelic targeting PCR screen showing that all *ACTC1* ^WT/MUT^ clones tested resulted in a 517bp product and were therefore heterozygous for *AAVS1* targeting. L – 1kb ladder; N – no template control; U – untargeted cell line; + - *AAVS1* biallelic positive control. ( **D**) Screening *MYH7* ^MUT/MUT^ clones using immunocytochemistry on differentiated hiPSC-CMs reveals a significant increase in R-GECO1.0 expression in the *MYH7* ^MUT/MUT^ Hom 2 clone. Mean ±SD, n = 3 technical replicates. One-way ANOVA with Tukey’s multiple comparison test, ** p ≤ 0.006; **** p < 0.0001. Scale bars = 50 µm.

The *AAVS1* locus, located within the first intron of *PPP1R12C* on chromosome 19 ( Figure 1A), is a well characterised safe harbour locus ^2^. Using the five lines above, we targeted a cassette containing R-GECO1.0 reporter and a puromycin resistance cassette, driven by the CAG promoter, into the *AAVS1* locus ( Figure 1A) ^24^. This was achieved by using a CRISPR-Cas9 nickase approach based on two sgRNAs. Nucleofection of the HCM-associated hiPSCs with the R-GECO1.0 construct, bidirectional sgRNAs and Cas9 D10A nickase plasmids produced puromycin-resistant clones. PCR-based screening and sequencing were used to examine the regions upstream ( Figure 1B) and downstream ( Figure 1C) of the insertion site, and hence identify clones that were successfully targeted in one or both alleles ( Figure 2B, C).

**Figure 3.**
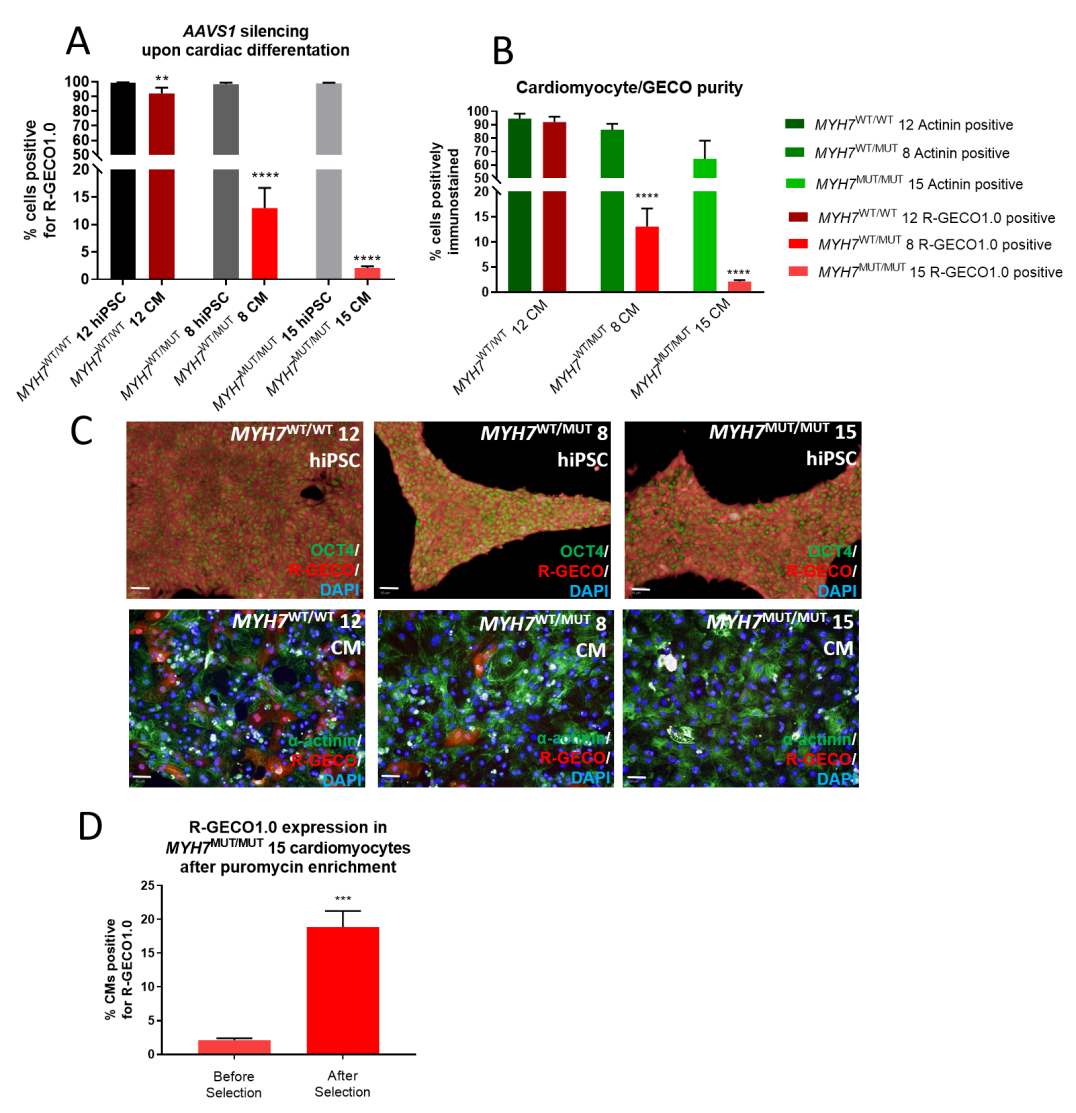
Changes in transgene expression during differentiation and antibiotic selection. ( **A**) Immunocytochemistry using an anti-RFP antibody to detect R-GECO1.0 expression shows a reduction in signal in *MYH7* ^WT/MUT^ 8 and *MYH7* ^MUT/MUT^ 15 cell lines upon differentiation from hiPSCs to hiPSC-CMs. Mean ±SD, n = 3 technical replicates. One-way ANOVA with Sidak’s multiple comparison test, ** p = 0.0015; **** p ≤ 0.0001. ( **B**) Percentage purity data for cardiomyocytes determined by alpha-actinin staining (green), for R-GECO1.0 by RFP staining (red). Mean ±SD, n = 3 technical replicates. One-way ANOVA with Sidak’s multiple comparison test, **** p ≤ 0.0001. ( **C**) Targeted hiPSC lines show variations in expression of R-GECO1.0 protein upon differentiation. hiPSC lines, identified by OCT4 staining (green, top row) show high R-GECO1.0 expression (red). Upon differentiation to cardiomyocytes, identified by α-actinin staining (green, bottom row), *MYH7* ^WT/MUT^ 8 and *MYH7* ^MUT/MUT^ 15 hiPSC-CMs show lower R-GECO1.0 expression (red). Scale bars = 50 µm. ( **D**) R-GECO1.0 expression in *MYH7* ^MUT/MUT^ 15 cardiomyocytes is significantly improved with three passages of hiPSC cell culture in 0.3 µg/ml puromycin and differentiation carried out in media supplemented with puromycin. Mean ±SD, n = 3 technical replicates. Unpaired t-test, *** p = 0.0003.

In addition, the *MYH7* ^WT/WT^ hiPSC line was used to introduce a HOXA9-T2A-mScarlet cassette driven by a doxycycline-inducible pTRE promoter into the *AAVS1* locus using the same CRISPR-Cas9 nickase approach ( Figure 1A). Both 5’ ( Figure 1D) and 3’ integration ( Figure 1E) to *AAVS1* was assessed using the same PCR genotyping approach on genomic DNA to identify successfully targeted clones.

### Variability in transgene expression at the *AAVS1* locus

In order to quantify R-GECO1.0 expression across the targeted clones, high content image analysis was used on hiPSCs that were dual-stained with anti-OCT4 for pluripotency and anti-RFP antibody, which identifies R-GECO1.0 ( Figure 2A) ^24^.

Transgene expression varied widely both between, and within, cell lines. The percentage of cells expressing R-GECO1.0 in *AAVS1*-targeted *ACTC1* ^WT/WT^ hiPSC clones ranged from a maximum of 96.4% to a minimum of 32.6% ( Figure 2A), and in isogenic mutant *ACTC1* ^WT/MUT^ hiPSC clones from 49.4% to 0%. Selected clones for the isogenic trio of *MYH7* ^WT/WT^, *MYH7* ^WT/MUT^ and *MYH7* ^MUT/MUT^ hiPSCs showed comparatively high R-GECO1.0 expression exceeding 73.09% in all cases, an important requirement for a more faithful comparison between lines ( Figure 2A).

This variability could not be explained by the incidence of biallelic targeting, as determined by PCR screening. Both alleles were targeted in *ACTC1* ^WT/WT^ clones three and six, which exhibited high R-GECO1.0 expression as hiPSCs of 91.1% and 85.4%, respectively. This was comparable with the 95.6% and 96.4% expression observed in the monoallelically targeted clones 11 and 12, respectively ( Figure 2A and 2B). Variability between clones was also seen upon differentiation, with a *MYH7* ^MUT/MUT^ Hom 2 clone showing 93.1% R-GECO1.0 expression as hiPSC-CMs, significantly greater than the 2.1% expression observed in the *MYH7* ^MUT/MUT^ Hom 3 clone (**** p = < 0.0001) ( Figure 2D).

Taken together, these results highlight that expression levels of transgenes seen in hiPSCs can vary significantly between *AAVS1*-targeted clones, which continued to be observed upon differentiation. Importantly, the level of variability could not be predicted and needed to be tested empirically.

### Transgene silencing upon mesoderm differentiation

Three *AAVS1*-targeted clones of each c. *MYH7* ^C9123T^ genotype were identified with greater than 98.3% R-GECO1.0 expression as hiPSCs ( Figure 3A and 3C) ^24^. However, upon differentiation to cardiomyocytes, R-GECO1.0 expression significantly reduced in the biallelically targeted *MYH7* ^WT/WT^ clone (** p = 0.0015). The monoallelically targeted *MYH7* ^WT/MUT^ and *MYH7* ^MUT/MUT^ clones experienced considerable silencing of R-GECO1.0 expression upon differentiation, with only 13.03% of *MYH7* ^WT/MUT^ hiPSC-CMs and 1.33% of *MYH7* ^MUT/MUT^ hiPSC-CMs expressing R-GECO1.0 (**** p < 0.0001) ( Figure 3A–C). Nevertheless, to enhance the utility of these lines, particularly the low expressing *MYH7* ^MUT/MUT^ clone, puromycin enrichment of the hiPSCs over three passages was used to significantly increase the number of R-GECO1.0 expressing hiPSC-CMs from 1.3% to 18.9% (*** p = 0.0003) ( Figure 3D). These results show that high transgene expression from the *AAVS1* locus as hiPSCs is not a guarantee of continued high expression upon differentiation, and points towards some extent of silencing upon cardiac differentiation.

**Figure 4.**
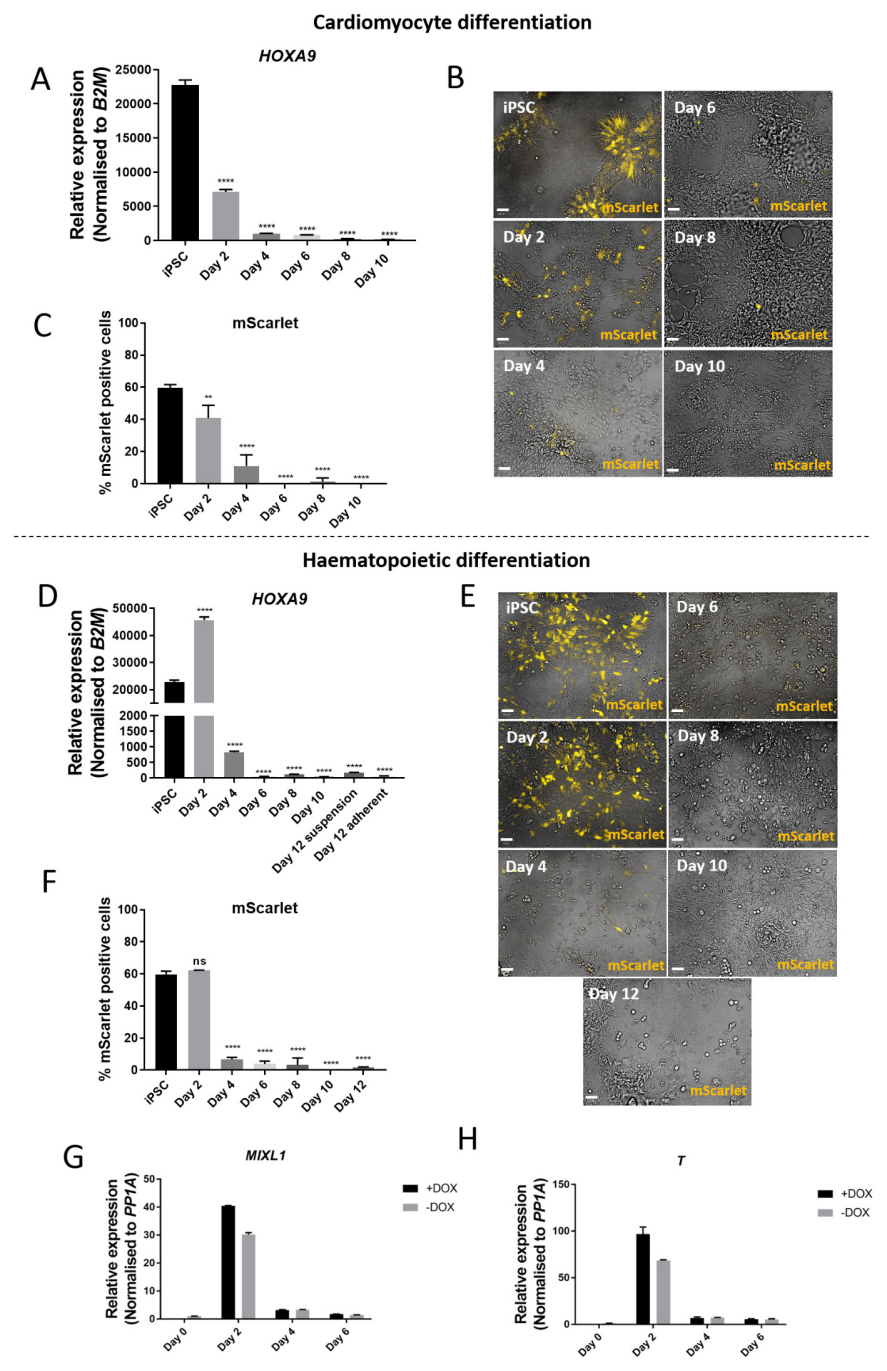
Differentiation towards cardiomyocytes or haematopoietic cells results in silencing of *AAVS1*-targeted transgene after mesoderm induction. ( **A**) qRT-PCR performed on hiPSCs targeted at the *AAVS1* locus with a doxycycline-inducible HOXA9-T2A-mScarlet construct undergoing cardiomyocyte differentiation. Relative expression to untargeted hiPSCs. Reduced expression of the transgene is observed from day two onwards. ( **B**) Live imaging of *AAVS1*-targeted hiPSCs undergoing cardiomyocyte differentiation at different timepoints. mScarlet expression peaks on day 0 and reduces throughout the differentiation, despite repeated doxycycline treatment. Scale bars = 50 µm. ( **C**) Quantification of mScarlet expression using high content image analysis at different timepoints during cardiomyocyte differentiation. ( **D**) qRT-PCR performed on hiPSCs targeted at the *AAVS1* locus with a doxycycline-inducible HOXA9-T2A-mScarlet construct undergoing haematopoietic differentiation. Relative expression to untargeted hiPSCs. Reduced expression of the transgene is observed from day four onwards. ( **E**) Live imaging of *AAVS1*-targeted hiPSCs undergoing haematopoietic differentiation at different timepoints. Scale bars = 50 µm. ( **F**) Quantification of mScarlet expression using high content image analysis shows peak expression on day two and reduced expression thereafter. Mean ±SD, n = 2 differentiations. One-way ANOVA with Dunnett’s multiple comparison test, ** p = 0.0036; **** p ≤ 0.0001. ( **G**, **H**) The mesoderm markers *MIXL1* and Brachyury ( *T*) show peak expression at day two of haematopoietic differentiation.

Next, we sought to investigate the time at which silencing occurs during differentiation. To do this, we used the *AAVS1*-targeted doxycycline-inducible HOXA9-T2A-mScarlet line and differentiated the hiPSCs towards either cardiomyocyte or haematopoietic fate. As expected, the addition of 1 µg/ml doxycycline every 48 hours induced expression of *HOXA9* and mScarlet during directed cardiac and haematopoietic differentiation. qRT-PCR analysis of *HOXA9* expression showed an increase of 22738-fold higher expression compared to untargeted hiPSC control on day 0 of cardiomyocyte differentiation ( Figure 4A) ^24^. However, *HOXA9* expression decreased thereafter so that by day 10, expression levels were only 175-fold greater than untargeted hiPSC control. Similarly, qRT-PCR analysis during haematopoietic differentiation revealed peak expression of HOXA9 occurring on day two, with 45666-fold greater expression than untargeted hiPSC control, decreasing thereafter to 64-fold expression on day 12 ( Figure 4D). These results were mirrored at the protein level, where early peak expression of mScarlet fluorescence occurred on day 0 of cardiomyocyte differentiation ( Figure 4B and 4C) and on day two of haematopoietic differentiation ( Figure 4E and 4F), decreasing at later timepoints of differentiation.

For the haematopoietic differentiation, expression of the key mesoderm markers *MIXL1* and Brachyury peaked on day two ( Figure 4G and 4H). This suggests that silencing of the *AAVS1* locus can occur immediately after mesoderm patterning. As a whole, these results show a progressive silencing of transgene expression as mesoderm differentiation progresses.

### *AAVS1*-targeted R-GECO1.0 expressing clones as a tool for *in vitro* disease modelling and drug screening

**Figure 5.**
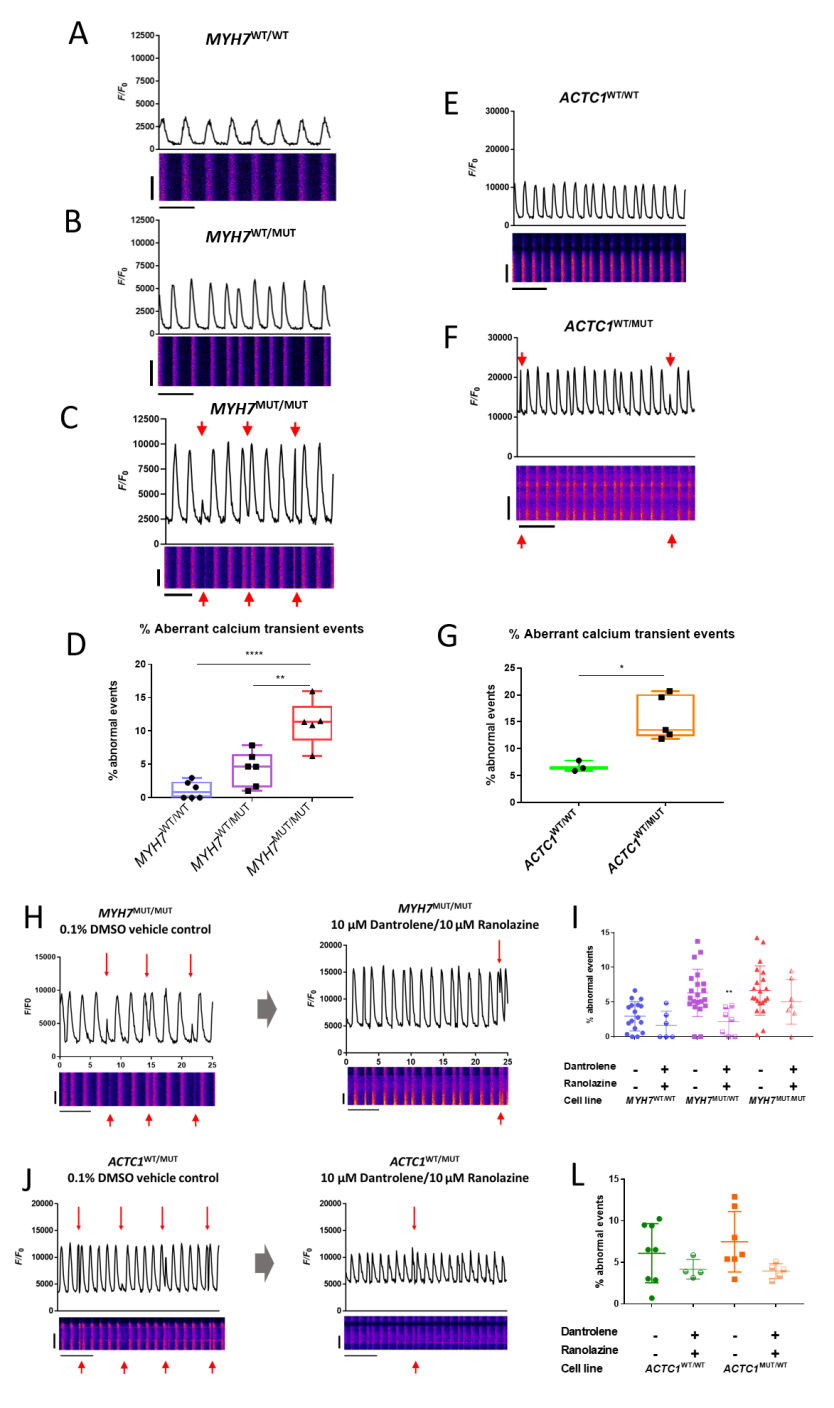
Functional application of *AAVS1*-targeted R-GECO1.0 expressing clones for *in vitro* disease modelling of HCM and phenotypic rescue with drug treatment. ( **A**– **C**) Representative 25-second confocal laser line scan traces and kymographs for isogenic trio of c. *MYH7* ^C9123T^ R-GECO1.0 expressing hiPSC-CMs at day 30. Abnormal Ca ^2+^ transient events (red arrows) increase in frequency with mutation load. x-axis scale bar = 20 µm, y-axis scale bar = 5 seconds. ( **D**) Ca ^2+^ transient event detection and quantification showing percentage of Ca ^2+^ transients deemed abnormal. Data presented as mean ±SD, n = 6 scans across three differentiations. Kruskal-Wallis test with Dunn’s multiple comparison test; ** p = 0.0018, **** p < 0.0001. ( **E**– **F**) Representative 25-second confocal laser line scan traces and kymographs for isogenic pair of c. *ACTC1* ^G301A^ R-GECO1.0 expressing hiPSC-CMs at day 30. Abnormal Ca ^2+^ transient events (red arrows) increase in frequency with mutation load. x-axis scale bar = 20 µm, y-axis scale bar = 5 seconds. ( **G**) Box-plot showing % of total Ca ^2+^ transient events detected deemed abnormal by event detection software. Data presented as mean ±SD, n = 5 scans from three differentiations. Unpaired t-test; * p = 0.0118. ( **H**– **L**) Representative line scans and event detection quantification showing a reduction in abnormal Ca ^2+^ transient events upon treatment with 10 µM ranolazine and 10 µM dantrolene compared to 0.1% DMSO vehicle control. Data presented as mean ±SD, n > 4 cells across minimum of two differentiations. Kruskal-Wallis test with Dunn’s multiple comparison test; ** p = 0.0068. x-axis scale bar = 20 µm, y-axis scale bar = 5 seconds.

Despite some obstacles due to unanticipated *AAVS1* silencing, isogenic R-GECO1.0 expressing clones in genetic backgrounds associated with HCM were successfully generated and used to image Ca ^2+^ transients using confocal laser line scan microscopy. As this technique involves assaying single cells, even poorly expressing clones were useful. By monitoring the fluctuation of R-GECO1.0 fluorescence over time, Ca ^2+^ transient traces could be generated for each line ( Figure 5A–C and 5E–F) ^24^. Despite some expected variability in spontaneous beat rate between wild-type hiPSC-CMs ( Figure 5A and 5E) ^29^, for both the c. *MYH7* ^C9123T^ and c. *ACTC1* ^G301A^ mutations, increasing mutation load resulted in an increased incidence of abnormal Ca ^2+^ transient events. *MYH7* ^WT/WT^ hiPSC-CMs only presented 1.1% aberrant Ca ^2+^ transient events, increasing to 4.33% in *MYH7* ^WT/MUT^ hiPSC-CMs, and further increasing to 11.2% in *MYH7* ^MUT/MUT^ hiPSC-CMs (p < 0.0001) ( Figure 5D). This represented a ten-fold increase in the occurrence of aberrant Ca ^2+^ transients in the homozygous mutant compared to isogenic wild-type control. Likewise, 15.65% of Ca ^2+^ transients in *ACTC1* ^WT/MUT^ hiPSC-CMs were calculated as being aberrant, compared to 6.7% (±0.6%) in *ACTC1* ^WT/WT^ isogenic control hiPSC-CMs (p = 0.0118) ( Figure 5G). This demonstrated the utility of the *AAVS1*-targeted R-GECO1.0 cell lines for *in vitro* disease modelling and phenotyping as a credible alternative to the use of animal models.

We then attempted to rescue the aberrant Ca ^2+^ transient phenotype in our HCM models with the use of a combination treatment of ranolazine, a late sodium channel blocker, and dantrolene, a ryanodine receptor antagonist. In combination at 10 µM with 24 hours incubation, these two drugs significantly reduced the frequency of aberrant Ca ^2+^ transient events in *MYH7* mutant hiPSC-CMs. In *MYH7* ^WT/MUT^ hiPSC-CMs aberrant Ca ^2+^ transient event frequency was reduced from 6.32% (±3.4%) in vehicle control to 2.18% (±1.9%) with drug treatment (p = 0.0068) ( Figure 5H–I).

These results show that abnormalities in Ca ^2+^ transients caused by the sarcomeric mutations c. *MYH7* ^C9123T^ or c. *ACTC1* ^G301A^ can be identified with *AAVS1*-targeted R-GECO1.0 expression, and this phenotype can be subsequently rescued with targeted pharmacological intervention aimed at reducing intracellular Na ^+^ and Ca ^2+^.

## Discussion

Precise integration of exogenous DNA into the genome is often performed by targeting a genomic ‘safe harbour’ locus that can tolerate gene insertion with few deleterious effects and limited transgene silencing. The *AAVS1* locus is a popular choice for targeted knock in of exogenous DNA ^16, 17^. This region of the genome is claimed to facilitate robust and persistent transgene expression ^11^, aided by flanking insulator regions ^10^. Here, we show variable success targeting the *AAVS1* locus with the genetically encoded calcium indicator R-GECO1.0 or a doxycycline-inducible HOXA9-T2A-mScarlet cassette using a CRISPR Cas9 nickase approach.

Variability in R-GECO1.0 expression between *AAVS1*-targeted clones as hiPSCs was observed, highlighting the importance of thorough screening of clones. Unsurprisingly, clones that had undergone biallelic targeting retained high R-GECO1.0 expression as hiPSCs, yet monoallelically targeted clones ranged from high expression to significantly reduced R-GECO1.0 expression. These incidences of low expression as hiPSCs may be due to clone-specific silencing, or some clones favouring expression from the untargeted allele. It has been shown that some genes within cells favour monoallelic expression ^30^. Indeed, our own studies using an antibody for the c. *ACTC1* ^G301A^ mutation have shown that cells heterozygous for the mutation only express mutant protein in ~50% of the population ^21^. These results highlight the heterogeneity that can exist between clones once they have been generated.

Even with the identification of *AAVS1*-targeted clones that exhibited robust R-GECO1.0 expression, there were instances of silencing upon differentiation to hiPSC-CMs. Transgene silencing at the *AAVS1* locus has previously been shown upon differentiation towards hepatocyte-like cells, with *de novo* methylation of the locus found to be responsible ^12^. However, the aforementioned report claims that this silencing effect is restricted to endoderm differentiations. With the use of the *AAVS1*-targeted HOXA9-T2A-mScarlet cell line, we show that in two mesoderm-specific differentiations towards cardiomyocytes and haematopoietic cells, silencing of expression occurs immediately after mesoderm specification and peak expression of *MIXL1* and *Brachyury*. This is in agreement with a recent report which demonstrated differential transgene methylation in *AAVS1*-targeted iPSC-derived myeloid cells ^13^. We therefore postulate that *AAVS1*-mediated silencing, likely as a result of *de novo* methylation, can occur upon differentiation to various germ layers, and therefore *AAVS1* cannot be considered a true safe harbour locus.

The choice of promoter may also play a role in *AAVS1*-mediated silencing, as a previous report has shown *AAVS1* silencing of eGFP expression when using the EF1a promoter that was overcome with the use of the stronger CAG promoter ^31^. This influenced our decision to opt for the CAG promoter over the EF1a promoter in our constructs. Indeed, the CAG promoter appears to exhibit some insulation from methylation of transgenes at the *AAVS1* locus ^12^. In addition, a recent report elegantly demonstrated that contextual silencing at the AAVS1 locus of iPSC-derived myeloid precursors can occur with varying efficiency depending on the chosen promoter ^13^.

When attempting to express two separate transgenes from the same cassette at the *AAVS1* locus, the choice of peptide cleavage sequence is also important. We observed inefficient peptide cleavage when using the P2A sequence, as determined by Western Blot (see *Extended data*) ^24^. This informed our choice of using the T2A or IRES sequences for translation of multicistronic cassettes in subsequent targeting constructs. It has been claimed that the P2A cleavage sequence is the most efficient self-cleaving peptide, followed by T2A and E2A, when used to cleave a bicistronic vector in three different human cell lines, including HeLa ^32^. We cannot reconcile this with our data, as co-expression of multicistronic cassette elements from the *AAVS1* locus in hiPSCs has been achieved using the IRES and T2A sequence, but not the P2A sequence.

Depending on the application of the targeted cell lines, some *AAVS1*-mediated silencing can be tolerated. For the R-GECO1.0 expressing lines, maintaining puromycin selection and using a single cell confocal laser line scan assay to study Ca ^2+^ transients meant that abnormalities in Ca ^2+^ handling could be identified in cell lines harbouring the c. *MYH7* ^C9123T^ or c. *ACTC1* ^G301A^ mutations. This represents an *in vitro* model of HCM that can offer an alternative to the use of animal models. Furthermore, this phenotype could be rescued with combination treatment with dantrolene and ranolazine ^20, 21^. However, the aim with the doxycycline-inducible HOXA9-T2A-mScarlet cell line was to modulate HOXA9 expression at different timepoints throughout haematopoietic differentiation. Clearly, the clone described herein is not suitable for this task.

In conclusion, one must carefully select *AAVS1*-targeted clones depending on their application due to the risk of transgene silencing upon differentiation. Other potential safe harbour loci, such as *CLYBL*, have been claimed to deliver five- to ten-fold higher fluorescent transgene expression than *AAVS1* ^6^. However, as silencing cannot be predicted, multiple clones must be thoroughly checked for expression level and chosen according to their application. Our results dispute the claims of robust and persistent transgene expression from *AAVS1* ^11^, and complements reports that show silencing at *AAVS1* upon differentiation to endoderm lineage ^12^, by showing similar silencing upon mesoderm differentiation.

## Data availability

### Underlying data

FigShare: Variable expression and silencing of CRISPR-Cas9 targeted transgenes identifies the AAVS1 locus as not an entirely safe harbour. https://doi.org/10.6084/m9.figshare.c.4573316.v3 ^24^

This project contains the following underlying data:

- Figure 1 – data (raw PCR integration gel images in TIF format; and sequencing chromatograms underlying Figure 1 as AB1 files)
- Figure 2 – data (raw immunocytochemistry images in PNG format; PCR gel images in TIF and PNG format; and XLS files containing immunocytochemistry expression quantification data underlying Figure 2)
- Figure 3 – data (raw immunocytochemistry images in PNG format; and XLS files containing expression quantification data underlying Figure 3)
- Figure 4 – data (raw live imaging images in PNG format; XLS files containing qPCR data underlying Figure 4)
- Figure 4 data – Raw qPCR data (XLS files containing raw qPCR data)
- Figure 5 – data (kymographs in PNG format; XLS files containing raw confocal laser line scan data underlying Figure 5)
- Figure 7 – data (raw PCR integration gel images in TIF format)

### Extended data

FigShare: Variable expression and silencing of CRISPR-Cas9 targeted transgenes identifies the AAVS1 locus as not an entirely safe harbour. https://doi.org/10.6084/m9.figshare.c.4573316.v3 ^24^

This project contains the following extended data:

- Figure 6. Western Blot to show incomplete cleavage of multicistronic cassette when using the P2A peptide cleavage sequence (TIF image file)
- Figure 7. PCR screening of hiPSC clones to check for AAVS1 integration (TIF image file)
- Figure 8. sgRNA design (TIF image file)
- Table 2.1. Guide 2 off-target locations (XLS file)
- Table 2.2 Guide 3 off-target locations (XLS file)
- Figure 9. Delta Ct representation of HOXA9 expression in AAVS1-targeted hiPSCs (TIF image file)
- Protocol for CRISPR Cas9 knock-in at the AAVS1 locus and subsequent screening (a step-by-step procedure for performing CRISPR Cas9 knock-in at the *AAVS1* locus of hiPSCs, followed by subsequent screening techniques in DOCX format)

Data are available under the terms of the Creative Commons Attribution 4.0 International license (CC-BY 4.0).
