# Supplementary material for: Variable expression and silencing of CRISPR-Cas9 targeted transgenes identifies the AAVS1 locus as not an entirely safe harbour
Source: F1000Res. 2020 Jul 10;8:1911. Originally published 2019 Nov 12. [Version 2] doi: 10.12688/f1000research.19894.2 (PMC7401084; doi:10.12688/f1000research.19894.2)
Supplement: Peer review history

# Peer review: Reviewer response for version 2

**DOI:** 10.5256/f1000research.26839.r67081

**Published:** 2020-07-30

**Authors:** Sara Howden

**Affiliations:** 1 Murdoch Children's Research Institute (MCRI), Melbourne, Vic, Australia

In this study, the authors clearly show that targeting transgenes to the "safe harbour" AAVS1 results in highly variable transgene expression in iPSCs, which is exacerbated even further following differentiation. This has important implications for researchers contemplating this locus for generating iPSC lines to stably express a transgene of interest (e.g fluorescent reporter, Cas9, transcription factor etc) The study appears well executed and their conclusions are well supported by their findings. One minor comment but certainly not a deal breaker, it would have been nice to see clones analysed by flow cytometry. This would not only give a very accurate picture of the number of cells expressing the given reporter but also a more accurate picture of the level of transgene expression (i.e median peak fluorescence) not only between clones but within clones. 

 Also, might be helpful if the authors indicate how they separate endogenous HOXA9 expression from exogenous (transgene) expression.

 All in all, a great little study with very useful data/implications for those in the field!

Is the work clearly and accurately presented and does it cite the current literature?

Yes

If applicable, is the statistical analysis and its interpretation appropriate?

I cannot comment. A qualified statistician is required.

Are all the source data underlying the results available to ensure full reproducibility?

Yes

Is the study design appropriate and is the work technically sound?

Yes

Are the conclusions drawn adequately supported by the results?

Yes

Are sufficient details of methods and analysis provided to allow replication by others?

Partly

Reviewer Expertise:

Gene editing, pluripotent stem cells, kidney differentiation

I confirm that I have read this submission and believe that I have an appropriate level of expertise to confirm that it is of an acceptable scientific standard.

# Peer review: Reviewer response for version 2

**DOI:** 10.5256/f1000research.26839.r66913

**Published:** 2020-07-10

**Authors:** Carolyn Carr

**Affiliations:** 1 Department of Physiology, Anatomy and Genetics, University of Oxford, Oxford, UK

No further comments

Is the work clearly and accurately presented and does it cite the current literature?

Yes

If applicable, is the statistical analysis and its interpretation appropriate?

Yes

Are all the source data underlying the results available to ensure full reproducibility?

Yes

Is the study design appropriate and is the work technically sound?

Yes

Are the conclusions drawn adequately supported by the results?

Yes

Are sufficient details of methods and analysis provided to allow replication by others?

Partly

Reviewer Expertise:

Cardiac physiology and cardiac stem cells for regeneration, disease phenotyping and drug testing

I confirm that I have read this submission and believe that I have an appropriate level of expertise to confirm that it is of an acceptable scientific standard.

# Peer review: Reviewer response for version 1

**DOI:** 10.5256/f1000research.21830.r61069

**Published:** 2020-03-23

**Authors:** Catherine M. Verfaillie, Yannan Fan

**Affiliations:** 1 Stem Cell Institute Leuven, Department of Development and Regeneration, KU Leuven, Leuven, Belgium; 2 KU Leuven, Leuven, Belgium

In this paper, the authors aim to investigate the effect of hypertrophic cardiomyopathy associated mutations *MYH7C ^9123T^* and *ACTC1 ^G301A^* on Ca ^2+^ transients using hiPSCs- derived cardiomyocytes.

 To this end, the authors established hiPSCs reporters by introducing a CAG promotor-controlled calcium indicator (R-GECO1.0) into the *AAVS1* locus through CRISPR/Cas9 nickase-mediated genome editing. Among 24 clones, 20 were found to successfully express the transgene with variation from about 0 to 99.5%. Upon differentiation to cardiomyocytes, even clones with high expression levels demonstrated significant silencing of the transgene to 13.03% or 1.33%. By creating also an *AAVS1* targeted doxycycline inducible HOXA9-T2A-mScarlet iPSCs reporter, the authors investigated the silencing over the time course during mesoderm lineage commitment. It was shown that both mRNA and protein levels of the transgenes were relatively high on day 2 and abruptly decreased from day 4 during both cardiomyocyte and hematopoietic differentiation.

 Despite the silencing of R-GECO1.0 in the *AAVS1* locus, Ca ^2+^ live image by confocal laser line scan microscopy at the single cell level detected abnormal Ca ^2+^ transients in cardiomyocytes derived from hiPSCs reporters harboring *MYH7C* or *ACTC1 *mutation *s *compared to wildtype or isogenic control. In addition, this abnormality could be rescued by pharmacological inhibiting intercellular level of Na+ and Ca ^2+^.

 This study shows that iPSC reporter line is of importance for disease modelling. This study presented an important issue in *AAVS1*-targeted transgene expression in iPSCs and mesoderm lineage although the mechanisms for the silencing are not clear. The variable expression and silencing in mesoderm differentiation shown here are in line with previous reports that the AAVS1 is not a true safe harbor for cells differentiated to hematopoietic cells (e.g. PMID: 31773990) and endoderm (e.g. PMID: 26455413); and, hence, the findings are not fully novel.

**Specific comments:**

1. The authors did not mention why for they compared wild-type ( *MYH7* ^WT/WT^), heterozygous ( *MYH7* ^WT/MUT^) and homozygous ( *MYH7* ^MUT/MUT^) for the c. *MYH7* ^C9123T^ mutation, but no homozygous mutant line for *ACTC1 ^G301A^*.
2. In Fig 2A, the expression of R-GECO in *ACTC1* ^WT/MUT^ clones (1/5 with about 50% cells) was lower than in *ACTC1* ^WT/WT ^clones. Is this chance or due to the mutation?
3. In Fig 2A, it would be better to show R-GECO in red and OCT4 in green to retain consistency with other images.
4. In Fig 3C, immunostaining in the upper images showed R-GECO in red while in the lower ones R-GECO is stained in green, which is difficult to follow.
5. It is interesting to see that puromycin enrichment of the iPSCs over 3 passages increased R-GECO1.0 expression in *MYH7* ^MUT/MUT^ 15, did the author also tried puromycin enrichment in *MYH7* ^WT/MUT^?
6. In the introduction, the authors mention that a doxycycline-inducible HOXA9-T2A-mScarlet cassette targeted in the *AAVS1* locus of hiPSCs is used for modulating HOXA9 during hematopoietic differentiation. However, this iPSC reporter is also used for cardiomyocyte differentiation (Fig 4A). It is unclear whether there is specific reason why this reporter line is used instead of *AAVS1*-CAG R-GECO iPSCs.
7. It is better to present RTqPCR result using a ∆Ct method for Fig. 4 as fold changes are confusing especially in the context of transgene expression (the biological relevance of fold change is unclear unless one knows the base line transcript levels before gene activation).
8. In Fig 6 - Western Blot (Extended data), to test the peptide cleavage of P2A and T2A, the authors state that P2A is less efficient than T2A, as NpHR expression (following a P2A) was barely visible. The authors should provide positive control to exclude that the antibody for NpHR did not work.
9. Southern blots should be performed to make sure that clones tested in this study were targeted in the corrected locus, and silencing was not due to random integration.

Is the work clearly and accurately presented and does it cite the current literature?

Partly

If applicable, is the statistical analysis and its interpretation appropriate?

Yes

Are all the source data underlying the results available to ensure full reproducibility?

Yes

Is the study design appropriate and is the work technically sound?

Yes

Are the conclusions drawn adequately supported by the results?

Partly

Are sufficient details of methods and analysis provided to allow replication by others?

Yes

Reviewer Expertise:

iPSC, differentiation, genome editing

We confirm that we have read this submission and believe that we have an appropriate level of expertise to state that we do not consider it to be of an acceptable scientific standard, for reasons outlined above.

# Response

**Published:** 2020-05-21

**Authors:** Jamie Bhagwan

**Affiliations:** The University of Nottingham, UK

**Dear Reviewer,**

**Thank you for your comments on our manuscript entitled *Variable expression and silencing of CRISPR-Cas9 targeted transgenes identifies the AAVS1 locus as not an entirely safe harbour.***

**We have endeavoured to respond to your comments and suggestions as outlined below in bold font.**

In this paper, the authors aim to investigate the effect of hypertrophic cardiomyopathy associated mutations *MYH7C* *^9123T^* and *ACTC1* *^G301A^* on Ca ^2+^ transients using hiPSCs- derived cardiomyocytes.

To this end, the authors established hiPSCs reporters by introducing a CAG promotor-controlled calcium indicator (R-GECO1.0) into the *AAVS1* locus through CRISPR/Cas9 nickase-mediated genome editing. Among 24 clones, 20 were found to successfully express the transgene with variation from about 0 to 99.5%. Upon differentiation to cardiomyocytes, even clones with high expression levels demonstrated significant silencing of the transgene to 13.03% or 1.33%. By creating also an *AAVS1* targeted doxycycline inducible HOXA9-T2A-mScarlet iPSCs reporter, the authors investigated the silencing over the time course during mesoderm lineage commitment. It was shown that both mRNA and protein levels of the transgenes were relatively high on day 2 and abruptly decreased from day 4 during both cardiomyocyte and hematopoietic differentiation.

Despite the silencing of R-GECO1.0 in the *AAVS1* locus, Ca ^2+^ live image by confocal laser line scan microscopy at the single cell level detected abnormal Ca ^2+^ transients in cardiomyocytes derived from hiPSCs reporters harboring *MYH7C* or *ACTC1 *mutation *s *compared to wildtype or isogenic control. In addition, this abnormality could be rescued by pharmacological inhibiting intercellular level of Na+ and Ca ^2+^.

This study shows that iPSC reporter line is of importance for disease modelling. This study presented an important issue in *AAVS1*-targeted transgene expression in iPSCs and mesoderm lineage although the mechanisms for the silencing are not clear. The variable expression and silencing in mesoderm differentiation shown here are in line with previous reports that the AAVS1 is not a true safe harbor for cells differentiated to hematopoietic cells (e.g. PMID: 31773990) and endoderm (e.g. PMID: 26455413); and, hence, the findings are not fully novel.

**We note the reviewer’s comments regarding novelty of the findings. However, the Klatt *et al*. paper (PMID: 31773990) was published after the submission of this manuscript. The Klatt *et al*. paper was first published online on 27 November 2019, whilst this manuscript was first published on 12 November 2019, so the variable expression and silencing upon hematopoietic differentiation was undocumented prior to the submission of this manuscript. Nonetheless, the Klatt *et al* paper adds weight to the notion of *AAVS1* silencing, and elegantly demonstrates the contextual methylation of *AAVS1*-targeted transgenes depending on the promoter inserted into the site. We also note the Luo *et al* 2014 paper (PMID: 24833591) which showed silencing of eGFP expression at the *AAVS1* site when using the EF1α promoter that was overcome by replacing it with the CAG promoter. Indeed, this paper informed our choice of promoter for the RGECO targeting construct.**

**The penultimate sentence of the third paragraph in the Introduction now reads:**

“However, some reports of DNA methylation dampening transgene expression in both hPSC-derived hepatocytes (Ordovás et al., 2015) and iPSC-derived myeloid progenitors (Klatt *et al*., 2020) raise questions on whether a ‘perfect’ safe harbour locus exists”

**The Discussion now reads:**

“Transgene silencing at the AAVS1 locus has previously been shown upon differentiation towards hepatocyte-like cells, with de novo methylation of the locus found to be responsible (Ordovás *et al*., 2015). However, the aforementioned report claims that this silencing effect is restricted to endoderm differentiations. With the use of the *AAVS1*-targeted HOXA9-T2A-mScarlet cell line, we show that in two mesoderm-specific differentiations towards cardiomyocytes and haematopoietic cells, silencing of expression occurs immediately after mesoderm specification and peak expression of *MIXL1* and *Brachyury*. This is in agreement with a recent report which demonstrated differential transgene methylation in *AAVS1*-targeted iPSC-derived myeloid cells (Klatt et al., 2020).”

**And**

“Indeed, the CAG promoter appears to exhibit some insulation from methylation of transgenes at the *AAVS1* locus (Ordovás *et al*., 2015). In addition, a recent report elegantly demonstrated that contextual silencing at the *AAVS1* locus of iPSC-derived myeloid precursors can occur with varying efficiency depending on the chosen promoter (Klatt et al., 2020).”

**The authors recognise and acknowledge that this phenomenon has been shown in endoderm and this is referred to in the manuscript.**

**The last paragraph of the Introduction contains the following sentence:**

“This suggests that silencing at the *AAVS1* locus is not limited to the endoderm lineage as previously described (Ordovás *et al*., 2015).

**The Discussion states that:**

“Our results dispute the claims of robust and persistent transgene expression from AAVS1, and complements reports that show silencing at AAVS1 upon differentiation to endoderm lineage (Ordovás *et al*., 2015)”

**Specific comments:**

1. The authors did not mention why for they compared wild-type ( *MYH7* ^WT/WT^), heterozygous ( *MYH7* ^WT/MUT^) and homozygous ( *MYH7* ^MUT/MUT^) for the c. *MYH7* ^C9123T^ mutation, but no homozygous mutant line for *ACTC1* *^G301A^*. **The authors apologise for not making this clearer in the text. The discrepancy between the range of genetically-engineered lines exhibiting the *MYH7*- or *ACTC1*- mutations is due to the source of the cells and their subsequent CRISPR/Cas9 targeting strategy. The *MYH7*-mutant hiPSC lines were generated by targeting the WT allele of unrelated healthy cell lines or origin (Mosqueira *et al.*, 2018), enabling the generation of heterozygous and homozygous clones. In contrast, the isogenic set of *ACTC1* lines was generated by genomic correction of the mutant allele of the starting hiPSC line derived from a heterozygous patient (using a donor vector containing the WT allele only) (Smith *et al*., 2018, Kondrashov *et al*., 2018). As such, homozygous mutant clones could not be generated by employing this strategy. Notwithstanding, the vast majority of HCM-causing mutations in the sarcomeric genes are heterozygous (Lopes et al, 2013), as homozygous mutations tend to be lethal. In particular, the mutations under study (p.R453C-βMHC and p.E99K-ACTC1) have never reported to be homozygous in patients, to the best of our knowledge. Therefore, the cellular models generated already encompass the patient-relevant genotypic status and as such provide an accurate characterization of HCM *in vitro*. Homozygous mutant lines were included to provide extra readout sensitivity for the phenotypic assays developed (as seen by more severe phenotypes in Figure 5).** **The first paragraph of the Results section now reads: “**Our overarching goal was to create two isogenic sets of hiPSC lines in order to study Ca ^2+^ handling in the context of *in vitro* models of the disease HCM. One isogenic trio comprised lines that were originally wild-type ( *MYH7* ^WT/WT^) and then CRISPR Cas9 edited to generate heterozygous ( *MYH7* ^WT/MUT^) and homozygous ( *MYH7* ^MUT/MUT^) mutants for the c. *MYH7* ^C9123T^ mutation (Mosqueira *et al*., 2018) . The other comprised a pair that were originally patient-derived heterozygous ( *ACTC1* ^WT/MUT^) for the c. *ACTC1* ^G301A^ mutation and CRISPR Cas9 corrected ( *ACTC1* ^WT/WT^) (Smith *et al*., 2018, Kondrashov *et al*., 2018).
2. In Fig 2A, the expression of R-GECO in *ACTC1* ^WT/MUT^ clones (1/5 with about 50% cells) was lower than in *ACTC1* ^WT/WT ^clones. Is this chance or due to the mutation? **We believe that this is unlikely to be due to the ACTC1 mutation but simply highlights the variability between different cell lines. Each cell line, and subsequently, each clone seemingly has varying levels of transgene silencing and extensive screening is therefore required, as we have performed.**
3. In Fig 2A, it would be better to show R-GECO in red and OCT4 in green to retain consistency with other images. **Agreed.** **The images in Figure 2A for *MYH7*^MUT/MUT^ clone 1 and *MYH7*^MUT/MUT ^clone 4 have now been pseudocoloured to match the other images in the panel.**
4. In Fig 3C, immunostaining in the upper images showed R-GECO in red while in the lower ones R-GECO is stained in green, which is difficult to follow. **Agreed. To retain consistency, the upper images in Figure 3C have been pseudocoloured so that R-GECO is always represented as red. Figure 2D and all accompanying graphs have also been changed to aid consistency.**
5. It is interesting to see that puromycin enrichment of the iPSCs over 3 passages increased R-GECO1.0 expression in *MYH7* ^MUT/MUT^ 15, did the author also tried puromycin enrichment in *MYH7* ^WT/MUT^? **Our aim was to have a high enough percentage of cardiomyocytes expressing R-GECO so that confocal line scan analysis was technically feasible. The *MYH7*^MUT/MUT^ 15 clone was originally enriched due to the extremely low level of cardiomyocyte R-GECO expression making it difficult to perform single cell analysis. In contrast, the 13.03% R-GECO expression in the *MYH7*^WT/MUT ^clone was sufficient to find R-GECO expressing cardiomyocytes in a single field of view. Therefore, antibiotic selection of the *MYH7*^WT/MUT ^line was not necessary. The purpose of Figure 3D is to provide a technical solution to lower levels or R-GECO expression, when required.**
6. In the introduction, the authors mention that a doxycycline-inducible HOXA9-T2A-mScarlet cassette targeted in the *AAVS1* locus of hiPSCs is used for modulating HOXA9 during hematopoietic differentiation. However, this iPSC reporter is also used for cardiomyocyte differentiation (Fig 4A). It is unclear whether there is specific reason why this reporter line is used instead of *AAVS1*-CAG R-GECO iPSCs. **We acknowledge the Reviewer’s point. As cardiomyocyte and haematopoietic differentiation share developmental stages (e.g., mesoderm induction), and given that HOXA9 was also shown to be expressed in early cardiac progenitor cells of the cardiac crescent (Behrens et al., 2013), we opted to perform both cardiomyocyte and haematopoietic differentiations using the HOXA9-T2A-mScarlet line. The introduction has been amended to clarify the overall purpose of the HOXA9-T2A-mScarlet line:**

“In addition, CRISPR Cas9 targeting of the AAVS1 locus was used to target a doxycycline-inducible HOXA9-T2A-mScarlet cassette into hiPSCs.  HOXA9 is a transcription factor regulated spatio-temporally during haematopoietic or cardiac development (Behrens *et al*., 2013) and the aim was to examine if controlled supplemental expression of HOXA9 resulted in more efficient production of mature cells.”

 

1. It is better to present RTqPCR result using a ∆Ct method for Fig. 4 as fold changes are confusing especially in the context of transgene expression (the biological relevance of fold change is unclear unless one knows the base line transcript levels before gene activation). **We believe that the ∆∆Ct method gives a better visualisation of the changes in transgene expression. The data in Figure 4 is presented as relative expression compared to *untargeted* iPSCs. Nevertheless, graphical presentation of ∆Ct values are provided in Extended data (Figure 9 Delta Ct representation of HOXA9 expression in AAVS1-targeted hiPSCs).**
2. In Fig 6 - Western Blot (Extended data), to test the peptide cleavage of P2A and T2A, the authors state that P2A is less efficient than T2A, as NpHR expression (following a P2A) was barely visible. The authors should provide positive control to exclude that the antibody for NpHR did not work. **While the authors agree that this could enhance the data, our initial assessment was based on the 60kDa protein being, in all likelihood, a fusion of ChR2 (30kDa) and NpHR (30kDa). The anti-eNpHR antibody (catalog #AS12 1851; Agrisera) has been validated for use in Western Blot applications but unfortunately is not provided with a positive control sample.**
3. Southern blots should be performed to make sure that clones tested in this study were targeted in the corrected locus, and silencing was not due to random integration. **The authors agree that integration of the cassette into the *AAVS1* locus is vital. This was tested using PCR genotyping from outside the arms of homology and into the cassette as shown in Figure 1. As an alternative to southern blots, we have now included original PCR screening gels as extended data (Figure 7 – PCR screening of hiPSC clones to check for AAVS1 integration). In addition, we have included details of the sgRNA design (Figure 8 – sgRNA design) and show that all off-target locations occur in introns (Table 2.1 Guide 2 off-targets and Table 2.2 Guide 3 off-targets).**

**References**

Behrens AN, Iacovino M, Lohr JL, et al.: Nkx2-5 mediates differential cardiac differentiation through interaction with Hoxa10. *Stem Cells Dev*. 2013;22(15):2211-2220. 23477547 10.1089/scd.2012.0611

Klatt D, Cheng E, Hoffmann D, et al.: Differential Transgene Silencing of Myeloid-Specific Promoters in the AAVS1 Safe Harbor Locus of Induced Pluripotent Stem Cell-Derived Myeloid Cells. Hum Gene Ther. 2020;31(3-4):199-210. 31773990 10.1089/hum.2019.194

Kondrashov A, Duc Hoang M, Smith JGW, et al.: Simplified Footprint-Free Cas9/CRISPR Editing of Cardiac-Associated Genes in Human Pluripotent Stem Cells. *Stem Cells Development*. 2018;27(6):391-404. 29402189 10.1089/scd.2017.0268 5882176

Lopes LR, Zekaviti A, Syrris P, et al.: Genetic Complexity in Hypertrophic Cardiomyopathy Revealed by High Throughput Sequencing. *J Med Genet*. 2013;50(4):228-39. 23396983 10.1136/jmedgenet-2012-101270

Luo Y, Liu C, Cerbini T, et al.: Stable enhanced green fluorescent protein expression after differentiation and transplantation of reporter human induced pluripotent stem cells generated by AAVS1 transcription activator-like effector nucleases. *Stem Cells Transl Med*. 2014;3(7):821–35. 24833591 10.5966/sctm.2013-0212 4073825

Mosqueira D, Mannhardt I, Bhagwan JR, et al.: CRISPR/Cas9 editing in human pluripotent stem cell-cardiomyocytes highlights arrhythmias, hypocontractility, and energy depletion as potential therapeutic targets for hypertrophic cardiomyopathy. *Eur Heart J.* 2018;39(43):3879–3892. 29741611 10.1093/eurheartj/ehy249 6234851

Ordovás L, Boon R, Pistoni M, et al.: Efficient Recombinase-Mediated Cassette Exchange in hPSCs to Study the Hepatocyte Lineage Reveals *AAVS1* Locus-Mediated Transgene Inhibition. *Stem Cell Reports.* 2015;5(5):918–31. 26455413 10.1016/j.stemcr.2015.09.004 4649136

Smith JGW, Owen T, Bhagwan JR, et al.: Isogenic Pairs of hiPSC-CMs with Hypertrophic Cardiomyopathy/LVNC-Associated ACTC1 E99K Mutation Unveil Differential Functional Deficits. *Stem Cell Reports.* 2018;11(5):1226–1243. 30392975 10.1016/j.stemcr.2018.10.006 6235010

# Peer review: Reviewer response for version 1

**DOI:** 10.5256/f1000research.21830.r56469

**Published:** 2019-11-29

**Authors:** Carolyn Carr

**Affiliations:** 1 Department of Physiology, Anatomy and Genetics, University of Oxford, Oxford, UK

There are two arms to this manuscript. The authors aimed to determine the reliability of introducing a transgene containing a calcium indicator (R-GECO1.0) or a fluorescent reporter (HOXA9-T2A-mScarlet) into the AAVS1 locus using CRISPR-Cas9 and then subsequently investigate the effect of mutations in *MYH7* or *ACTC1 *on calcium handling.

 Initial experiments showed that although the AAVS1 could be targeted successfully in 20/24 clones, the expression of the transgene was highly variable. Furthermore, on differentiation of the more successfully transfected clones to cardiomyocytes, the transgene was silenced with almost complete removal of expression of the fluorescent reporter. A similar degree of silencing was observed during haematopoetic differentiation.

 Using the mScarlet reporter the authors investigated the timecourse of downregulation and found that with both cardiomyocyte and haematopoetic differentiation, increased transgene expression was observed at day 2 but then decreased substantially over time, at both the mRNA and protein level.

 Nevertheless, using puromycin selection, it was possible to isolate single cardiomyocytes expressing the R-GECO reporter and these were used to show that mutations in both *MYH7* and *ACTC1 *resulted in abnormal calcium transient events which could be corrected in part using ranolazine and dantrolene.

 This is a valuable piece of work which demonstrates shortfalls in what was expected to be a fairly reliable transfection protocol and that the AAVS1 locus is not as foolproof a site for transfection as may have been thought. The addition of the calcium transient measurements is interesting in that it shows that something can be rescued from such a large body of work, but it does come as rather an afterthought in the manuscript.

 I have a few minor comments to aid clarity:

- The introduction needs to be expanded somewhat. The paragraph on modulating HOXA9 is very brief and the rationale needs to be explained. In the results section this appears to be merely a way of monitoring transgene expression, but the short paragraph in the introduction implies some sort of mechanistic approach.
- It is not entirely clear to me what the difference is between the data shown in Fig 2D and 3B apart from different clones.
- In the section on HOXA9-T2A targeting it says in results that doxycycline was administered every 48 hours. This is not mentioned in the methods or the extended information. The authors state that transgene induction decreased after day 2. Was this reduction despite further addition of doxycycline?
- In figure 5, the timing on the x axis is given in parts H and I but not in other plots. In some others there is a bar but this is not explained. Is the scale the same throughout? If so, some comment should be made as to why the wild type MYH7 cells have a slower beat rate than the ACTC1 wild type cells. The same formatting should be used on all the graphs.

Is the work clearly and accurately presented and does it cite the current literature?

Yes

If applicable, is the statistical analysis and its interpretation appropriate?

Yes

Are all the source data underlying the results available to ensure full reproducibility?

Yes

Is the study design appropriate and is the work technically sound?

Yes

Are the conclusions drawn adequately supported by the results?

Yes

Are sufficient details of methods and analysis provided to allow replication by others?

Partly

Reviewer Expertise:

Cardiac physiology and cardiac stem cells for regeneration, disease phenotyping and drug testing.

I confirm that I have read this submission and believe that I have an appropriate level of expertise to confirm that it is of an acceptable scientific standard.

# Response

**Published:** 2020-05-21

**Authors:** Jamie Bhagwan

**Affiliations:** The University of Nottingham, UK

**Dear Reviewer,**

**Thank you for your comments on our manuscript entitled *Variable expression and silencing of CRISPR-Cas9 targeted transgenes identifies the AAVS1 locus as not an entirely safe harbour.***

**We have endeavoured to respond to your comments and suggestions as outlined below in bold font.**

 There are two arms to this manuscript. The authors aimed to determine the reliability of introducing a transgene containing a calcium indicator (R-GECO1.0) or a fluorescent reporter (HOXA9-T2A-mScarlet) into the AAVS1 locus using CRISPR-Cas9 and then subsequently investigate the effect of mutations in *MYH7* or *ACTC1 *on calcium handling.

 Initial experiments showed that although the AAVS1 could be targeted successfully in 20/24 clones, the expression of the transgene was highly variable. Furthermore, on differentiation of the more successfully transfected clones to cardiomyocytes, the transgene was silenced with almost complete removal of expression of the fluorescent reporter. A similar degree of silencing was observed during haematopoetic differentiation.

 Using the mScarlet reporter the authors investigated the timecourse of downregulation and found that with both cardiomyocyte and haematopoetic differentiation, increased transgene expression was observed at day 2 but then decreased substantially over time, at both the mRNA and protein level.

 Nevertheless, using puromycin selection, it was possible to isolate single cardiomyocytes expressing the R-GECO reporter and these were used to show that mutations in both *MYH7* and *ACTC1 *resulted in abnormal calcium transient events which could be corrected in part using ranolazine and dantrolene.

 This is a valuable piece of work which demonstrates shortfalls in what was expected to be a fairly reliable transfection protocol and that the AAVS1 locus is not as foolproof a site for transfection as may have been thought. The addition of the calcium transient measurements is interesting in that it shows that something can be rescued from such a large body of work, but it does come as rather an afterthought in the manuscript.

 I have a few minor comments to aid clarity:

- The introduction needs to be expanded somewhat. The paragraph on modulating HOXA9 is very brief and the rationale needs to be explained. In the results section this appears to be merely a way of monitoring transgene expression, but the short paragraph in the introduction implies some sort of mechanistic approach.
**For the purposes of this paper the HOXA9 model is simply used a tool to monitor transgene expression. However, its overall purpose in the context of haematopoiesis is now described in the introduction for completeness. The penultimate paragraph of the Introduction now reads:**
  “In addition, CRISPR Cas9 targeting of the AAVS1 locus was used to target a doxycycline-inducible HOXA9-T2A-mScarlet cassette into hiPSCs.  HOXA9 is a transcription factor regulated spatio-temporally during haematopoietic or cardiac development (Behrens *et al*., 2013) and the aim was to examine if controlled supplemental expression of HOXA9 resulted in more efficient production of mature cells.”
- It is not entirely clear to me what the difference is between the data shown in Fig 2D and 3B apart from different clones.
**Yes, we concede that these experiments are similar. However, Fig 2D is intended to show variability within an isogenic set of clones differentiated to cardiomyocytes, whereas Fig 3B is intended as a comparison between isogenic sets of clones and complements the data in Fig 3A.**
- In the section on HOXA9-T2A targeting it says in results that doxycycline was administered every 48 hours. This is not mentioned in the methods or the extended information. The authors state that transgene induction decreased after day 2. Was this reduction despite further addition of doxycycline?
**Yes, this reduction did occur despite further addition of doxycycline every 2 days. In addition to text in the results and the figure legend for Figure 3, the methods have now been clarified with a sentence in the ‘Live imaging of mScarlet’  section which now reads:**
  “HOXA9 and mScarlet expression was induced with the addition of 1 µg/ml doxycline every 48 hours.”

 

- In figure 5, the timing on the x axis is given in parts H and I but not in other plots. In some others there is a bar but this is not explained. Is the scale the same throughout? If so, some comment should be made as to why the wild type MYH7 cells have a slower beat rate than the ACTC1 wild type cells. The same formatting should be used on all the graphs.
**The authors apologise for the error. The x axis scale bar relates to time (5 seconds) and the y-axis scale bar relates to the length of the laser line drawn across the cell to perform the confocal line scan. All panels now contain both scale bars and the legend for Figure 5 has been corrected to include this information. The methods have also been clarified to state that these line scans are performed on spontaneously beating cardiomyocytes, hence their slightly varied beat rate. The end of the first paragraph of the ‘confocal analysis’ methods sections now reads: **
**“**Line-scan images were taken every 75 milliseconds, with a pixel dwell time of 4.12 µsec, for a total of 4000 cycles resulting in a five minute scan. CMs were kept at 37°C and 5% CO 2 and allowed to spontaneously beat throughout data acquisition. **”**
**Differences in beat rate and action potential duration between healthy hiPSC-CMs are common, as** **previously** **reviewed** **(** **Sala** ***et al*** **.** **, 2017). This further advocates the need for isogenic lines in order to ensure th** **at the impact o** **f the mutation studied is accurately investigated.** **The fourth sentence of the section entitled “AAVS1-targeted R-GECO1.0 expressing clones as a tool for in vitro disease modelling and drug screening” now reads:**
  “Despite some expected variability in spontaneous beat rate between wild-type hiPSC-CMs (Figure 5A and 5E) (Sala *et al*., 2017), for both the c. *MYH7* C9123T and c. *ACTC1* G301A mutations, increasing mutation load resulted in an increased incidence of abnormal Ca ^2+^ transient events.”

**References**

 Behrens AN, Iacovino M, Lohr JL, et al.: Nkx2-5 mediates differential cardiac differentiation through interaction with Hoxa10. *Stem Cells Dev*. 2013;22(15):2211-2220. 23477547 10.1089/scd.2012.0611

 Sala L, Bellin M, Mummery CL.: Integrating cardiomyocytes from human pluripotent stem cells in safety pharmaology: Has the time come? *Br J Pharmacol*. 2017;174(21):3749-3765. 27641943 10.1111/bph.13577
